# Targeting immune checkpoint pathways overcomes tumor-induced CD8+ T cell suppression

**DOI:** 10.1186/2051-1426-2-S3-P119

**Published:** 2014-11-06

**Authors:** Lukas W Pfannenstiel, Brian R Gastman

**Affiliations:** 1 Cleveland Clinic, Cleveland, OH, USA

## 

The immune system has the potential to be a powerful tool to destroy tumors. However despite ample evidence of anti-tumor immune responses in many patients, as well as years of immunotherapy development, truly effective immune-based therapies remain out of reach. We have previously shown that short-term *in vitro *co-culture of human T cells with human-derived tumor cell lines of various origins can induce the gain of senescence-like features in both CD4+ and CD8+ T cells, particularly the loss of CD27/CD28 expression. We found this tumor-induced senescence does not involve activation/proliferation, and also results in the gain of a potent suppressive function in *in vitro *suppression assays. In subsequent studies, we found that IL-7 could protect T cells from CD27/CD28 loss and acquisition of suppressive function and that this process is highly dependent on the expression of the anti-apoptotic protein Mcl-1.

In the current study we sought to determine whether a similar process could not only be found in mice, but also in tumor-resident T cells (TIL) collected from human patient specimens. We show that the process of tumor-induced dysfunction also induces the expression of PD-1 in both human and mouse T cells *in vitro*, and that tumor-exposed mouse T cells are also capable of suppressive function. Similar to published reports, we find tumor-resident human CD8+ TIL to have significant losses in CD27/CD28 expression, and that loss of these markers is accompanied by the gain in PD-1 expression, as well as other known checkpoint inhibitors, especially Tim-3. In mice, TIL also highly express PD-1, Tim-3, and Lag-3 but similar to *in vitro *studies, do not lose expression of CD27 and CD28. Using cell sorting to isolate CD3+ CD8+ PD-1+ Tim-3+ T cells from both mouse and human tumors, we find that this population is able to suppress autologous or syngeneic responder T cells in *ex vivo *proliferation assays. We further show that use of antibody blockade of PD-1 and Tim-3 signaling *in vitro *and *in vivo *prevents the development of suppression. Ongoing studies will examine the signaling mechanisms responsible, especially by evaluating the role of Mcl-1 expression in generation of dysfunctional CD8 T cells *in vivo*. These studies demonstrate that tumor-derived factors can induce suppressive CD8+ T cells, and that blockade of negative regulators of T cell function can help prevent this process, indicating an additional benefit to the clinical use of these immunotherapies.

